# Ill-lighting syndrome: prevalence in shift-work personnel in the anaesthesiology and intensive care department of three Italian hospitals

**DOI:** 10.1186/1745-6673-4-6

**Published:** 2009-03-27

**Authors:** Ilaria Morghen, Maria Cristina Turola, Elena Forini, Piero Di Pasquale, Paolo Zanatta, Teresa Matarazzo

**Affiliations:** 1 Anaesthesiology and Intensive Care Department, S. Anna University Hospital, C.so Giovecca 203, 44100, Ferrara, Italy; 2 Psychiatry Department, S. Anna University Hospital, C.so Giovecca 203, 44100, Ferrara, Italy; 3 Health Statistics Service, S. Anna University Hospital, C.so Giovecca 203, 44100, Ferrara, Italy; 4 Anaesthesia and Intensive Care Department, Rovigo Hospital, Viale 3 Martiri, 140, 45100, Rovigo, Italy; 5 Anaesthesia and Intensive Care Department, Treviso Regional Hospital, Viale Vittorio Veneto, 18, 31100, Treviso, Italy; 6 Anaesthesiology and Intensive Care Department, S. Anna University Hospital, C.so Giovecca 203, 44100, Ferrara, Italy

## Abstract

**Background:**

Light is one of the most important factors in our interaction with the environment; it is indispensable to visual function and neuroendocrine regulation, and is essential to our emotional perception and evaluation of the environment. Previous studies have focussed on the effects of prolonged anomalous exposure to artificial light and, in the field of work-related illness. Studies have been carried out on shift-work personnel, who are obliged to experience alterations in the physiological alternation of day and night, with anomalous exposure to light stimuli in hours normally reserved for sleep. In order to identify any signs and symptoms of the so-called ill-lighting syndrome, we carried out a study on a sample of anaesthesiologists and nurses employed in the operating theatres and Intensive Care Departments of three Italian hospitals. We measured the subjective emotional discomfort (stress) experienced by these subjects, and its correlation with environmental discomfort factors, in particular the level of lighting, in their workplace.

**Methods:**

We used a questionnaire developed by the Scandinavian teams who investigated Sick-Building Syndrome, that was self-administered on one day in the environments where the degree of illumination was measured according to UNIEN12464-1 regulations.

**Results:**

Upon comparison of the types of exposure with the horizontal luminance values (lux) measured (< 700 lux, between 1000–1500 lux, > 1500 lux) and the degree of stress reported, (Intensive Care: mean stress = 55.8%, high stress = 34.6%; Operating Theatres: mean stress = 51.5%, high stress = 33.8%), it can be observed that the percentage of high stress was reduced as the exposure to luminance was increased, although this finding was not statistically significant.

**Conclusion:**

We cannot share other authors' enthusiasm regarding the effects on workers well-being correlated to the use of fluorescent lighting. The stress level of our workers was found to be more heavily influenced by their familial and working conditions, irrespective of the ambient light stimulus.

## Background

Light is one of the most important factors in our interaction with the environment; it is indispensable to visual function and neuroendocrine regulation, and it is essential to our emotional perception and evaluation of the environment. Previous studies have demonstrated its effects on the psyche and also its therapeutic role: Berson, in 2002, documented the existence of a retinal photoreceptor linked to the supra-chiasmatic nucleus. This receptor has been ascribed a role in the transmission of neuronal transmission arising from light stimuli to the pineal gland, seat of the biological clock which presides over the regulation of the circadian system via the retinal hypothalamic pathway [1].

Control of the biological clock and the release of several important hormones (among which: cortisol, the stress hormone, and melatonin, the sleep hormone) are governed by the alternation of light and dark. Thus, exposure to light has important repercussions on human health and behaviour. A role in the regulation of the sleep/waking pattern, mood, body temperature and physical and cognitive performance has been attributed to daily and seasonal variations in light [2].

Recent studies have focussed on the effects of prolonged anomalous exposure to artificial light, both in outdoor and indoor environments, on alterations in the principal neuroendocrine mediators and on potential pathological effects such as: increased of risk of carcinogenesis, metabolic disorders (in particular obesity and diabetes), cardiovascular disease, acceleration of the aging process and alterations in regulation of the immune system [3,4]. Many other studies have examined the emotional value of light and its role in the treatment of mood disturbances [5,6].

Regarding work-related diseases, studies have been carried out on shift workers exposed to alterations in the normal day/night pattern, and thus to abnormal levels of intense light stimuli in the time usually reserved for sleep [7].

Does the so-called ill-lighting syndrome exist?

Begeman identifies the aetiology as an insufficient exposure to indoor light, with repercussions on workers' health and performance [8].

As recent research has demonstrated the physical characteristics of light act differently in determining visual and circadian photobiological functions of the retina.

The circadian system does not respond to the patterns of quantity, spectrum, distribution, time or duration of exposure to light which determine visual performance, but to the global sum of these criteria which penetrates the retina [9]. So it was necessary to study the characteristics of the spaces where examined workers operate. The anaesthesiologists and nurses employed in the operating theatres and Intensive Departments are shift workers that operate for prolonged exposure under fluorescent lighting. The aim of this study is to investigate if this job condition can affect the health workers and to identify signs and symptoms of an emotional discomfort (stress), that form part of an ill-lighting syndrome.

## Methods

### Participants

Observation was carried out on doctors and nurses from the Anaesthesia and Intensive Care Departments of three hospital, Ferrara, Rovigo and Treviso, in northern Italy. We measured subjective emotional discomfort (stress) and its correlation with environmental discomfort factors in the workplace, in particular the level of lighting. These shift workers operate in identical conditions of dress and posture, for prolonged periods of time, up to 12 hours per day, in environments lacking windows and therefore access to natural light.

**Figure 1:**
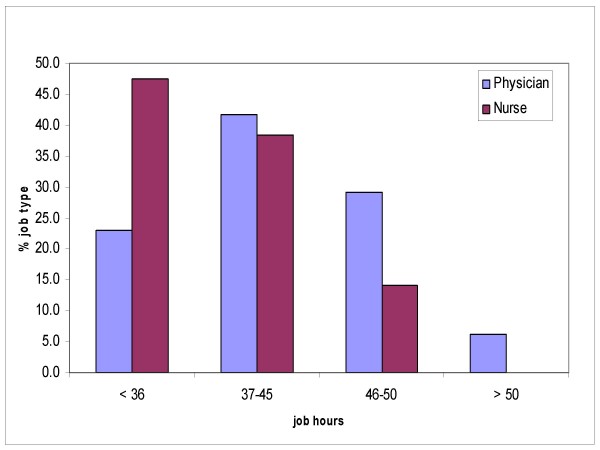
**Working hours by job type**.

The sample consisted of 134 workers, 35 (26,7%) males, 96 (73,2%) females, (3 were missing). 43% of males were between 46 and 55 years of age, 48% of females were between 36 and 45 years old. Forty eight (38%) were doctors, 78 (62%) were nurses, (8 were missing). Seventy one (53%) from Ferrara hospital, 32 (24%) from Rovigo hospital and 31 (23%) from Treviso hospital. The doctors have an average of 12,3 years (SD 9,6) of work experience, and the nurses an average of 14,3 years (SD 7,9). The medical staff studied carry out their professional duties in operating theatres and Intensive Care wards exposed solely to artificial light during their, on the whole, 37–45 hours of service per week for the doctors and 36 hours for the nurses (Figure 1).

### Setting

It was necessary to identify the characteristics of the spaces where the medical staff studied operate according to the regulation governing artificial illumination, the planning criteria of the illumination equipment and the distribution of light sources.

Measurement of illumination was carried out as per UNIEN12464-1 regulations [10]. The work environments in question lacked windows and were furnished with compact ultra-white fluorescent lights with a colour temperature of 3800 K and chromatic yield index of 96 Ra.

Based on the measurements of illumination obtained, the participants were divided into 3 exposure groups: < 700 lux, 1000–1500 lux, and > 1500 lux. Those in the Rovigo Hospital were exposed to less than 700 lux throughout the 24 hours of service whereas in Ferrara employees work in conditions of between 700 and 2000 lux. and finally in Treviso the light in the working environment was found to be between 1000 and 1500 lux.

### Procedure

In order to measure the level of stress experienced and emotional repercussion of light stimuli, a descriptive cross-sectional study was conducted on shift workers in environments lacking windows and therefore access to natural light.

We utilised a questionnaire inspired by Scandinavian research into Sick-Building Syndrome.

We modified Andersson's MM Questionnaire, combining it with the Stockholm Indoor Environment Questionnaire [11,12], and introduced items relating to the familial situation and job of the interviewee. We also included data regarding sleep, appetite and fatigue disturbances from the QIDS-SR16 (Quick Inventory of Depressive Symptomatology Self-report) [13], combining the scales in a single marker of emotional state, which we termed stress. The stress scale was divided into three levels: none, medium and high, based on the items relating to alterations in physical strength, sleep patterns and appetite, and to greater work-related irritability. Level 'none' was assigned when the worker gave all negative responses, 'high' when all affirmative responses were obtained, and 'medium' in all other cases. The questionnaire was self-administered on one day in the environments where the illumination was measured. The test subjects were not informed of the main objective of the study.

Validity and reliability: we used singly valid questionnaires. The Italian version was prepared by translation and retranslation. The questionnaire used in this survey was an administrative pilot. It's going to be retested on the same sample, during the same period of the year and in the same conditions as the first test given, to investigate the reliability.

### Statistical Analysis

Means and percentages were used for descriptive purposes. The chi-square test was used to compare qualitative variables, and statistical significance was defined as P < 0.05. SAS and SPSS (Statistical Analysis System, Software Products for Statistical Solutions) were used for statistical analysis.

The relationship between the following variables was considered by applying logistic regression analysis: weekly hours of work, professional qualifications (type of job), professional autonomy, perception of illumination, duration of exposure to natural light, familial responsibilities and level of stress.

## Results

The degree of stress reported were: Intensive Care, mean stress = 55.8%, high stress = 34.6%; Operating Theatres: mean stress = 51.5%, high stress = 33.8%).

**Figure 2:**
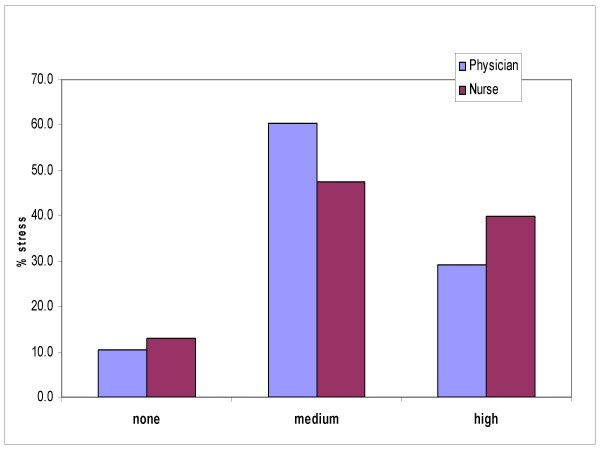
**Level of stress by job type**.

**Figure 3:**
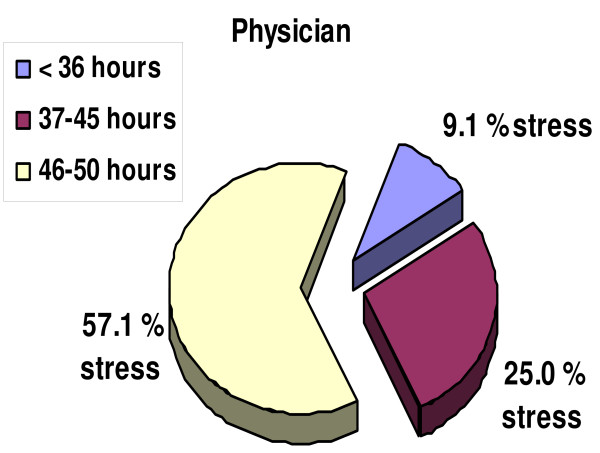
**Physicians' stress levels per length of working week**.

**Figure 4:**
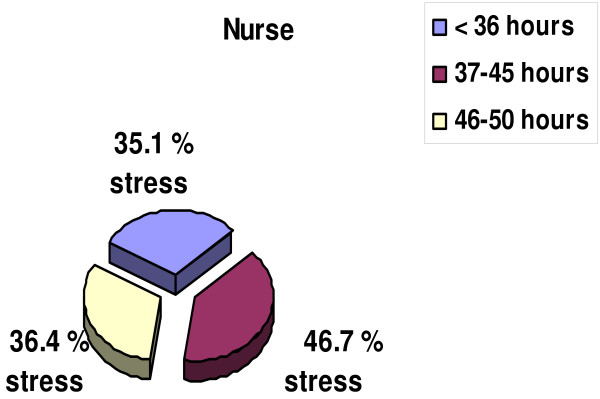
**Nurses' stress levels per length of working week**.

Comparing the number of hours worked per week by the doctors and nurses, it emerged that, although the former group worked longer hours, the latter tended to report higher levels of stress (Figure 2, 3); the doctors who worked from 46 to 50 hours per week reported stress levels of 57% and the nurses who worked from 37 to 45 hours per week reported stress levels of 46%, a higher amount of stress per hour. (Figure 4). The physicians declared a far higher capacity to influence their work flow (autonomy) than the nurses (62 vs. 38), with a statistical significance of p = 0.01 (Table 1).

**Table 1:** Possibility to influence work flow for physicians and nurses

	**Autonomy**			
	**Yes, often**	**Yes, sometimes**	**total**	**% Yes, often**
**Physician**	30	18	48	62.5
**Nurse**	29	47	76	38.2
**Total**	59	65	124	

Chi = 6.04 DF = 1 p = 0.01; Missing n = 10

**Figure 5:**
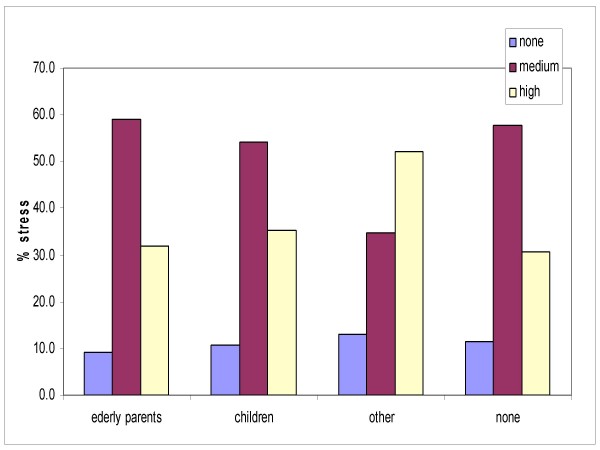
**Stress levels and familial responsibilities**.

**Figure 6:**
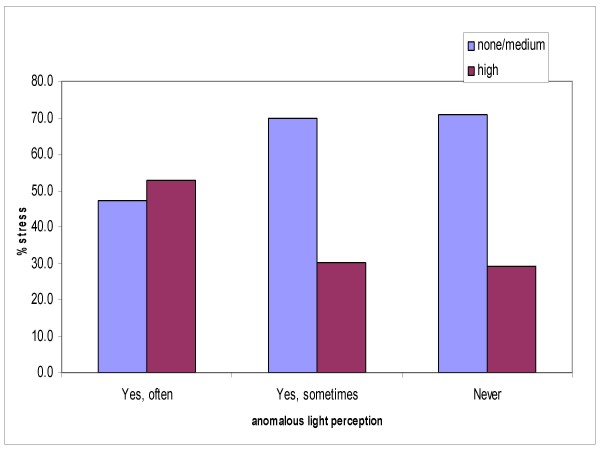
**Stress level and anomalous light perception**.

Among the familial responsibilities, those other than the care of elderly parents or children, such as the care of a pet, in particular a dog, had a tendency to elevate stress levels (Figure 5), which were comparable to those reported by workers who were discomforted by the artificial light in the operating theatres and Intensive Care departments (Figure 6).

**Figure 7:**
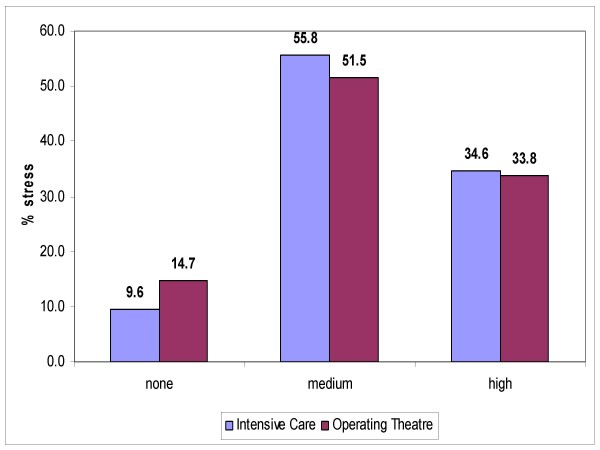
**Percentage stress in Intensive Care and operating theatre personnel**.

**Figure 8:**
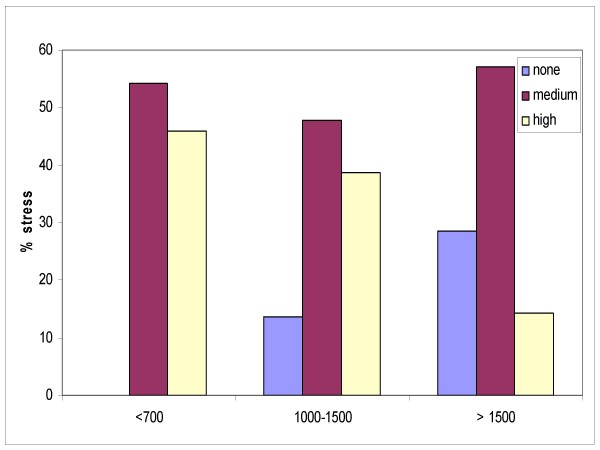
**Percentage of stress by exposure in lux**.

In order to verify the hypothesis that artificial light affects workers' mood, we carried out a logistic regression analysis using the stress index as the dependant variable (Figure 7), and how independent variables the types of exposure with the horizontal luminance (lux) (< 700 lux, between 1000–1500 lux, > 1500 lux), weekly hours of work, professional qualification (type of job). It can be observed that the percentage of high stress diminishes as the exposure to lux increases, although this reduction was not found to be statistically significant (Table 2, Figure 8).

**Table 2:** Stress level and exposure in lux

**stress**	**<700**	**1000–1500**	**> 1500**	**total**
**none**	0	6	4	10
**medium**	13	21	8	42
**high**	11	17	2	30
**total**	24	44	14	82

Chi = 8.8 DF = 4 p n.s.; Missing n = 52

## Discussion

The influence of light on human health, its role in regulation of the circadian rhythm, and its therapeutic applications in seasonal mood disorders have been described in numerous articles [2,6,14]. In studies on shift workers who experience overexposure to artificial light at night, an alteration in normal psychological and circadian behavioural rhythms have been revealed [3]. In our study we measured the subjective emotional discomfort and its correlation with environmental stimuli in the workplace, in particular the level of illumination.

In 2001, Veich and McColl disputed the validity of an effect of exposure to artificial light generated by fluorescent lights on development and psychological processes. More recently, the role of light in mood regulation of the circadian cycle and its psychological effects [1,3] has been the subject of rigorous studies aimed at finding correlations between the impact of light and colour on mood [6].

Küller explored the effect of illumination on workers subject to variations in stimuli in relation to their collocation in different seasons and latitudes. He found that the perception of light as anomalous affected the mood of the population, but this effect was not confirmed when the illumination was measured in objective terms. In our study we measured subjective emotional discomfort (stress) and its correlation with environmental discomfort factors in the workplace in particular the lighting level. The singularity of our study, focussed on identifying states of emotional disturbance in the Emergency Department personnel of three Italian hospitals, is the homogeneity of the sample. This consisted of public health workers who operate under the same environmental conditions; they carry out their duties in operating theatres and Intensive Care units, and are confined to closed, air-conditioned environments with a lack of natural light, which is substituted by the artificial variety in the form of fluorescent lights. Their clothing, their posture, the procedures they carry out (anaesthesiological assistance in a variety of surgical operations on critical patients), and the structural characteristics of the environments are all similar, differing only in the level of luminance measured.

Data on the levels of illumination in the workplace were obtained by measurements carried out according to European regulations, which indicate the quantitative and qualitative parameters of illumination in the workplace necessary to provide the workers with adequate visual comfort and performance. The subjects in our study manifested a medium-high level of stress, which, confirms the findings by Küller, showing no correlation with the objective ambient light measured. Also analogous to Küller's study, a tendency to increased stress was found in the subjects who reported a perceived light-caused discomfort, in terms of excessive glare or darkness.

Regarding the other items, it was observed that although the doctors work longer hours, the nurses reported a higher level of stress. It can be hypothesized that this is linked to the fact that the doctors have more autonomy at work, having decision-making powers that the nurses do not. Another characteristic of our survey is taking into consideration stress from familial conditions. The level of stress in our workers was found to be influenced by working and familial conditions, rather than ambient lighting stimuli.

Limitations of this study are due to small size of the sample and the need to improve the questionnaires reliability and validity. In the future we want to retest the same sample.

## Conclusion

Despite the limitations of the study, we cannot share in the enthusiasm of some authors, and the electronics industry, regarding the effects on well-being correlated to the use of fluorescent lamps [15]. The level of stress in our workers was found to be influenced by familial and working conditions, irrespective of ambient lighting stimuli.

## Abbreviations

QIDS-SR16: Quick Inventory of Depressive Symptomatology Self-report; Lux: horizontal luminance values; SAS: Statistical Analysis System; SPSS: Software Products for Statistical Solutions

## Competing interests

The authors declare that they have no competing interests.

## Authors' contributions

IM conceived the work, analyzed the data and collaborated in writing the article. MCT conceived the work, collected and analyzed the data and collaborated in writing the article. EF advised in all stages of the undertaking, analyzed the data and collaborated in writing the article. PDP analyzed the data and collaborated in writing the article. PZ collected data and collaborated in writing the article. TM analyzed the data and advised in all stages of the undertaking. All authors have read and approved the final manuscript.
